# Anxiety disorders are associated with reduced bone mineral density in men: Findings from the Geelong Osteoporosis Study

**DOI:** 10.1111/acps.13563

**Published:** 2023-05-08

**Authors:** Gregory Roebuck, Michael Mazzolini, Mohammadreza Mohebbi, Julie A. Pasco, Amanda L. Stuart, Malcolm Forbes, Michael Berk, Lana Williams

**Affiliations:** ^1^ The Institute for Mental and Physical Health and Clinical Translation (IMPACT), School of Medicine, Barwon Health Deakin University Geelong Victoria Australia; ^2^ Phoenix Australia – Centre for Posttraumatic Mental Health, Department of Psychiatry University of Melbourne Parkville Victoria Australia; ^3^ Melbourne Medical School, Faculty of Medicine, Dentistry and Health Sciences University of Melbourne Parkville Victoria Australia; ^4^ Biostatistics Unit Deakin University Geelong Victoria Australia; ^5^ Department of Medicine – Western Health University of Melbourne St Albans Victoria Australia; ^6^ Department of Epidemiology and Preventive Medicine Monash University Melbourne Victoria Australia; ^7^ Department of Psychiatry University of Melbourne Parkville Victoria Australia; ^8^ Orygen, The National Centre of Excellence in Youth Health, and the Florey Institute for Neuroscience and Mental Health, Department of Psychiatry University of Melbourne Parkville Victoria Australia

**Keywords:** anxiety, bone mineral density, depression, osteoporosis, psychiatric disorders

## Abstract

**Objective:**

Certain psychiatric disorders, including depression, appear to impact adversely on bone health. Anxiety disorders are highly prevalent but few studies have examined their effects on bone tissue. This study investigated the effect of anxiety disorders on bone mineral density (BMD).

**Methods:**

This prospective cohort study used data from the Geelong Osteoporosis Study. Participants were women and men aged ≥20 years randomly selected from the electoral roll and followed up for a mean of 14.7 and 11.0 years, respectively. Participants were assessed for a lifetime history of an anxiety disorder using the Structured Clinical Interview for DSM‐IV‐TR. BMD in the lumbar spine and femoral neck was measured using dual‐energy x‐ray absorptiometry.

**Results:**

Eight hundred and ninety women and 785 men participated in the study. Adjusting for sociodemographic, biometric and lifestyle factors, medical comorbidities and medication use, anxiety disorders were associated with reduced BMD at the lumbar spine (partial *η* ^2^ = 0.006; *p* = 0.018) and femoral neck (partial *η* ^2^ = 0.006; *p* = 0.003) in men. These associations became non‐significant when men with a history of comorbid mood disorders were excluded from the analysis. There was no significant association between anxiety disorders and BMD in women (*p* ≥ 0.168).

**Conclusions:**

Anxiety disorders are associated with reduced BMD in men. This effect may be mediated by comorbid depression.

**Significant outcomes**

- A lifetime history of a DSM‐IV‐TRanxiety disorder was associated with lower BMD at the lumbar spine and femoralneck in men (partial η^2^ = .06 for both associations).
- These associations may have been mediated by comorbid depression since they became non‐significant when participants with comorbid mood disorders were excluded from the analysis.
- Anxiety disorders were notassociated with differences in BMD in women.

**Limitations**

- Inquiring about past psychiatric symptoms may be associated with a recall bias.
- Defining the healthy group toinclude only participants with no history of a DSM‐IV‐TR psychiatric disorder may have inflated the differences between the anxiety disorders group and this group.
- The effects of some potential confounders (such as, for example, vitamin D levels) were not adjusted for.

## 1 INTRODUCTION

Osteoporosis is a bone disease characterised by reduced bone mass, microarchitectural deterioration of bone tissue and increased susceptibility to fracture.^1^ According to the World Health Organization definition, it is present when bone mineral density (BMD) is at least 2.5 standard deviations (SD) below the mean for a young, healthy reference population.^2^ Osteoporosis is highly prevalent in older adults.^3^ In the United Kingdom (UK), approximately one in two women and one in five men aged over 50 years will experience a fracture during their lifetime.^4^ Osteoporotic fractures are associated with extremely poor health outcomes.^5^, ^6^ Approximately one‐third of those who suffer an osteoporotic hip fracture die within the following year and only 30%–40% regain their premorbid level of functioning.^7^ As the global population ages, the burdens imposed by osteoporosis on patients, caregivers and health systems will increase significantly.^8^

Certain psychiatric disorders may impact negatively on bone health. Unipolar depression, bipolar disorder, schizophrenia and anorexia nervosa have all been reported to be associated with reduced BMD.^9^, ^10^, ^11^, ^12^ Apart from anorexia nervosa, the strongest evidence exists for unipolar depression. A 2016 meta‐analysis that pooled the results of 21 studies involving 1842 participants with depression and 17,401 controls found that depression was associated with lower BMD at the lumbar spine, femur and total hip.^12^ The composite weighted mean effect sizes ranged from small (Cohen's *d* = −0.14 for the total hip) to small‐to‐moderate (Cohen's *d* = −0.34 for the femur). The association between depression and reduced BMD persists after adjustment for a wide range of potential confounders, including age, body mass index (BMI), smoking, alcohol use, exercise levels, educational attainment, income and medical conditions known to affect bone health such as diabetes mellitus, hypertension and the metabolic syndrome.^13^

The mechanisms underlying the association between depression and lower BMD are unknown. Hypercortisolaemia is an established risk factor for osteoporosis and it has been hypothesised that increased glucocorticoid secretion may contribute to reduced BMD in depressed persons.^14^ Other proposed mechanisms include increased catecholamine secretion, increased secretion of pro‐inflammatory cytokines, increased oxidative stress, reduced vitamin D levels and the effects of antidepressant medications.^14^, ^15^, ^16^ There is some evidence that antidepressant medications are associated with reduced BMD,^17^ although a recent meta‐analysis failed to find a relationship between antidepressant use and BMD in women.^18^ Finally, the association between depression and reduced BMD may be mediated by risky health behaviours such as poor dietary intake or medication non‐adherence.

Anxiety disorders are the most prevalent psychiatric disorders globally, with an estimated 12‐month prevalence of 11.6%.^19^ They include generalised anxiety disorder, social anxiety disorder, panic disorder, agoraphobia and specific phobia. They are highly comorbid with depressive disorders and appear to share aetiological factors with these disorders.^20^, ^21^ Despite the high prevalence of anxiety disorders, only a small number of studies have explored their relationship with BMD. Two small studies of post‐menopausal women found that anxiety symptoms were associated with reduced BMD cross‐sectionally.^22^, ^23^ A large population‐based study in Norway found that anxiety symptoms were associated with lower BMD cross‐sectionally in men but not in women.^24^ Finally, a Taiwanese study found that individuals with an anxiety disorder diagnosis in a national health insurance database had a higher incidence of osteoporosis than matched controls.^25^ Existing studies have had several important limitations. First, they have used self‐report measures of anxiety symptoms rather than assessment by a clinician using a structured diagnostic instrument, the gold standard for psychiatric diagnosis. Secondly, they have failed to adjust for the effects of obvious potential confounders. Thirdly, they have been cross‐sectional or, in the case of the Norwegian study, have had a follow‐up period that may have been inadequate to detect any effects of anxiety disorders on the rate of change in BMD over time.

The aim of the current study was to investigate prospectively the relationship between clinician‐diagnosed anxiety disorders and BMD in women and men controlling for potential confounders. Given the established association between depression and reduced BMD, we hypothesised that anxiety disorders would be associated with lower BMD and an increased rate of bone loss over time in both sexes.

## 2 MATERIAL AND METHODS

### 2.1 Study population

This prospective cohort study used data from the Geelong Osteoporosis Study (GOS). The GOS is a population‐based cohort study that was launched in 1993 with the aim of investigating the epidemiology of osteoporosis in Australia. Since its commencement, the aims of the GOS have expanded to include investigating the relationship between mental illness and physical health. Women were recruited to the GOS between 1993 and 1997 and men were recruited between 2001 and 2006. Participants were drawn from the Barwon Statistical Division in Victoria, Australia. They were aged 20 years or older. An age‐stratified random sampling method was used, with 12 strata for each sex. Prospective participants were selected at random from the electoral roll and sent a personalised invitation to participate in the study by mail. A total of 2390 women were invited to participate, of whom 1938 were eligible and 1494 agreed to participate, representing participation among those eligible of 77%. A new sample of 246 women listed as aged 20–29 years on the 2005 electoral roll was recruited between 2006 and 2008. A total of 3273 men were invited to participate, of whom 2296 were eligible and 1540 agreed to participate, representing participation among those eligible of 67%. Reasons for ineligibility included death, having moved out of the study region, being uncontactable and being incapable of providing informed consent. Details regarding the methodology of the GOS, the characteristics of the study population and retention rates have been reported elsewhere.^26^

### 2.2 Procedure

Participants in the GOS were assessed at baseline and thereafter at regular time intervals. In this study, data were used from the baseline, 10‐year and 15‐year assessments for women and the baseline, 5‐year and 15‐year assessments for men. Clinical, biometric and self‐report data were collected at each assessment. Participants provided written, informed consent to their participation in the study. The study was approved by the Barwon Health Human Research Ethics Committee.

### 2.3 Measures

At each assessment, areal BMD at the lumbar spine (L2–L4) and femoral neck was measured using dual‐energy X‐ray absorptiometry (DXA). Weight and height were measured using electronic scales and a Harpenden wall‐mounted stadiometer. Participants also completed self‐report questionnaires that asked about demographic and lifestyle factors, including physical activity levels, smoking, diet and alcohol intake, medical history and current medication use. Physical activity levels were rated on a seven‐point Likert‐type scale from 1 (very active) to 7 (bedfast). Smoking status was dichotomised as current smoker or non‐smoker. Daily alcohol intake and calcium intake were calculated using a validated dietary questionnaire.^27^ Data regarding medical history were used to calculate participants' scores on the Charlson Comorbidity Index (CCI), a measure of medical comorbidity.^28^ Socioeconomic status was assessed by calculating participants' scores on the Index of Relative Socio‐Economic Disadvantage (IRSD) (for women) and Index of Socio‐Economic Advantage and Disadvantage (IRSAD) (for men).^29^ The IRSD and IRSAD both range on a five‐point Likert‐type scale from 1 (most disadvantaged) to 5 (least disadvantaged or most advantaged).

### 2.4 Psychiatric assessment

The Structured Clinical Interview for the text revision of the fourth edition of the Diagnostic and Statistical Manual of Mental Disorders (DSM‐IV‐TR), Non‐Patient Edition (SCID‐I/NP) was used to assess participants for a lifetime history of a DSM‐IV‐TR anxiety disorder. The anxiety disorders in DSM‐IV‐TR include panic disorder, agoraphobia, specific phobia, social phobia, obsessive–compulsive disorder (OCD), post‐traumatic stress disorder (PTSD), generalised anxiety disorder (GAD), anxiety disorder due to a general medical condition, substance‐induced anxiety disorder and anxiety disorder not otherwise specified. Participants were also assessed for a lifetime history of a mood disorder. The SCID‐I/NP is a clinician‐administered semi‐structured interview intended to yield diagnoses consistent with DSM‐IV‐TR.^30^ It was not administered at baseline but was administered at the 10‐year and 15‐year time points for women and the 5 and 15‐year time points for men. For the diagnosis of anxiety disorders, the inter‐rater reliability of the SCID‐I/NP for DSM‐IV‐TR ranges from fair (*κ* = 0.60 for agoraphobia) to excellent (*κ* = 0.83 for social phobia).^31^ Interviews were performed by clinicians with postgraduate qualifications in psychology who underwent training supervised by a psychiatrist that involved use of live and videotaped interviews, as recommended by First et al.^30^ The exposed group, or ‘anxiety disorders group’, included any participants who had a lifetime history of a DSM‐IV‐TR anxiety disorder according to the SCID‐I/NP at any time point. For participants in this group, age of onset was used to determine whether their anxiety disorder was present at the time of their baseline assessment. The unexposed group, or ‘healthy group’, included all participants who had no lifetime history of an Axis I psychiatric disorder according to the SCID‐I/NP at any time point.

### 2.5 Statistical analysis

Baseline differences between the anxiety disorders group and healthy group were explored. For continuous variables, an independent‐samples *t* test was performed for normally distributed data and a Mann–Whitney *U* test was conducted for non‐normally distributed data. For frequencies, the chi‐square test of independence was used. To explore the relationship between anxiety disorders and BMD, we performed a multivariable linear regression analysis using the method of generalised estimating equations (GEE) to account for the longitudinal nature of the study. The regression models included the nominal anxiety disorders group as the exposure of interest, time as a within‐subjects variable and continuous lumbar spine or femoral neck BMD as dependent variables. The following time‐updating variables were included as factors or covariates in the models: age at baseline, height, weight, IRSD/IRSAD, physical activity levels, smoking status, daily alcohol intake, daily calcium intake, CCI and use of adrenal steroid hormones, gonadal hormones, thyroid hormones or antithyroid agents, agents that affect calcium or bone metabolism and antidepressants. Interaction effects between the anxiety disorders group and each of the included variables were tested for significance and included in the model if significant. The presence of a significant interaction effect for group and time, group × time, was treated as indicating that anxiety disorders were significantly related to the rate of change in BMD over time. For each variable or interaction included in the model, the partial *η* ^2^ was calculated to provide a measure of effect size. Consistent with Cohen's recommendations, the following cut‐offs were used to interpret values for partial *η* ^2^: 0.01, small effect size; 0.06, medium effect size; and ≥0.14, large effect size.^32^ Given that there are well‐established sex differences in both the prevalence of anxiety disorders and BMD, data for women and men were analysed separately.^33^ For significant findings, an exploratory subgroup analysis was performed in which participants with a lifetime history of a comorbid mood disorder were excluded from the anxiety disorders group. The aim of this exploratory subgroup analysis was to investigate whether the relationship between anxiety disorders and BMD was mediated by the established relationship between depression and reduced BMD. The threshold for statistical significance was set at 0.05. Data were collected and managed using REDCap electronic data capture tools hosted at the University Hospital Geelong (Barwon Health). Analyses were performed using IBM SPSS Statistics version 28.

## 3 RESULTS

### 3.1 Baseline characteristics of the anxiety disorder and healthy groups

**FIGURE 1:**
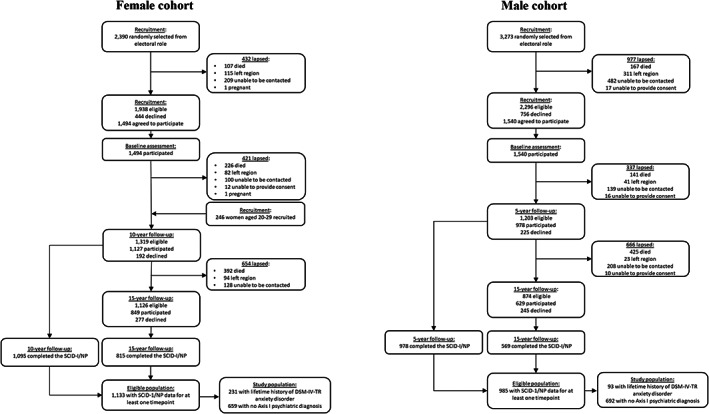
Participant flow diagrams for women and men.

**FIGURE 2:**
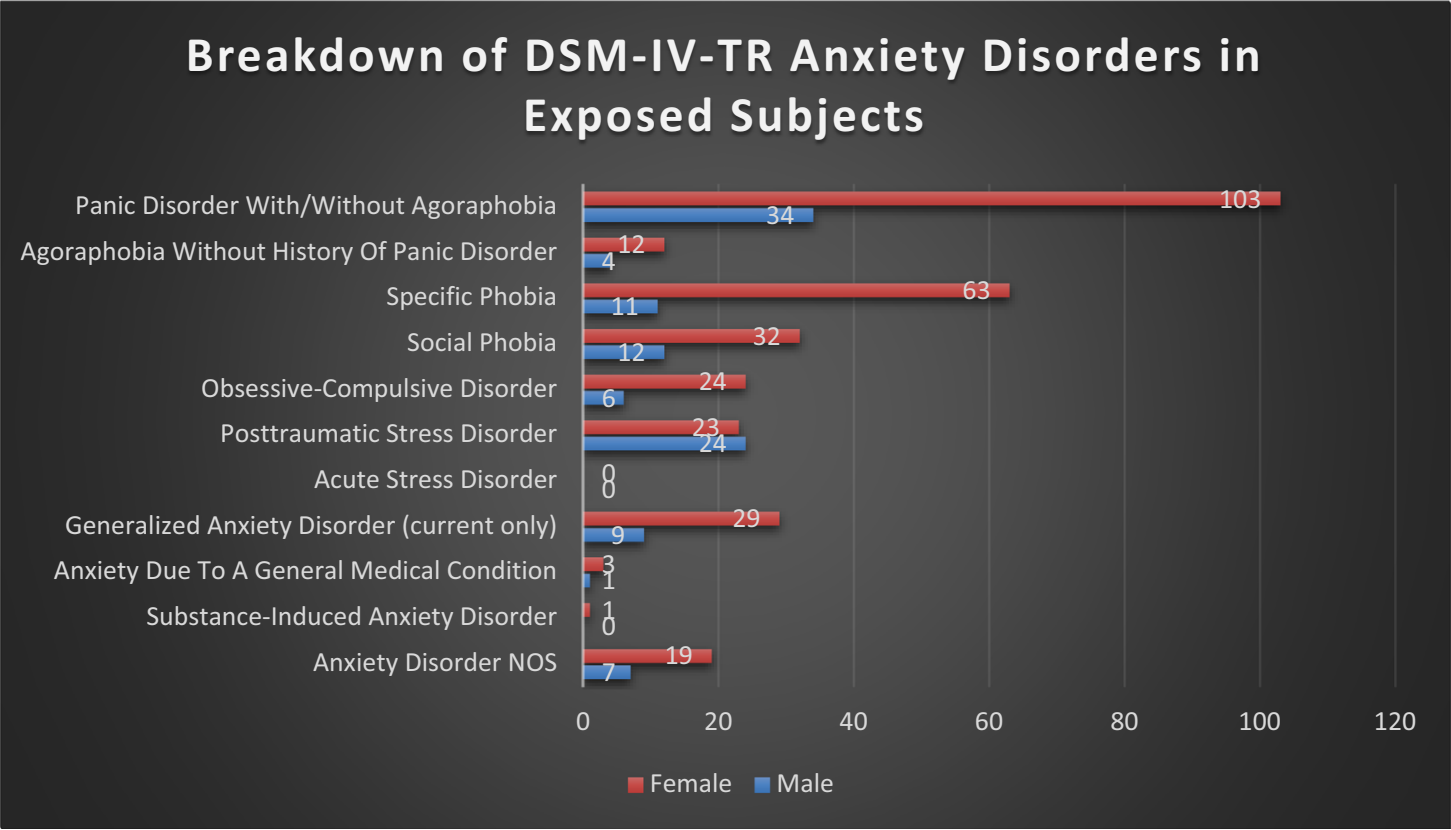
Numbers of participants with specific DSM‐IV‐TR anxiety disorders according to sex. This bar chart shows the numbers of participants who met criteria for specific DSM‐IV‐TR anxiety disorders according to sex. Panic disorder with agoraphobia and panic disorder without agoraphobia are combined into a single category in the chart. For GAD, only participants with a current history of this disorder are represented as the SCID‐I/NP for DSM‐IV‐TR does not assess for a lifetime history of GAD.

A total of 890 women and 785 men met the inclusion criteria and were included in the study. In the female cohort, there were 231 women in the anxiety disorders group and 659 women in the healthy group. In the male cohort, there were 93 men in the anxiety disorders group and 692 men in the healthy group. Participants were followed over a mean (SD) of 14.7 (3.2) years for women and 11.0 (4.0) years for men. Figure 1 shows participant flow diagrams for the study. Figure 2 shows the numbers of participants who met criteria for each of the different DSM‐IV‐TR anxiety disorders according to sex. For both sexes, panic disorder was the most common anxiety disorder. Table 1 sets out the baseline characteristics of the anxiety disorders group and healthy group according to sex. Women in the anxiety disorders group were younger, more likely to smoke and more likely to use antidepressant medication at baseline compared to the healthy group. They also had higher BMD at both the lumbar spine and femoral neck. Men in the anxiety disorders group were younger, taller, more likely to smoke, more likely to use antidepressant medication and had lower BMD at the lumbar spine at baseline compared to the healthy group.

**TABLE 1:** Baseline characteristics of the anxiety disorders and healthy groups for women and men.

Variable	Women		Men	
	Anxiety disorders group *n* (%)^a^	Healthy group *n* (%)^a^	Anxiety disorders group *n* (%)^a^	Healthy group *n* (%)^a^
Total *n* (%)	231 (26.0%)	659 (74.0%)	93 (11.8%)	692 (88.2%)
Age (years) median (IQR)	36 (28)*	45 (33)*	46 (19)*	57 (29)*
Height (cm) *M* (SD)	162.3 (6.0)	161.0 (6.1)	176.7 (5.4)*	174.6 (7.0)*
Weight (kg) *M* (SD)	69.2 (15.8)	68.8 (13.7)	84.7 (12.5)	82.8 (13.8)
Socioeconomic status				
Quintile 1 (most disadvantaged)	38 (22.4%)	91 (16.8%)	16 (17.2%)	114 (16.5%)
Quintile 2	35 (20.6%)	104 (19.2%)	22 (23.7%)	140 (20.2%)
Quintile 3	34 (20.0%)	112 (20.7%)	10 (10.8%)	135 (19.5%)
Quintile 4	28 (16.5%)	112 (20.7%)	19 (20.4%)	149 (21.5%)
Quintile 5 (least disadvantaged or most advantaged)	35 (20.6%)	122 (22.6%)	26 (28.0%)	154 (22.3%)
Physical activity levels				
1 (Very active)	28 (16.5%)	65 (12.0%)	16 (17.2%)	140 (20.2%)
2 (Active)	113 (66.5%)	345 (63.8%)	59 (63.4%)	415 (60.0%)
3 (Sedentary)	25 (14.7%)	119 (22.0%)	18 (19.4%)	131 (18.9%)
4 (Limited activity)	4 (2.4%)	11 (2.0%)	0 (0.0%)	6 (0.9%)
5 (Inactive)	0 (0.0%)	1 (0.2%)	0 (0.0%)	0 (0.0%)
6 or 7 (Chair/bedridden or bedfast)	0 (0.0%)	0 (0.0%)	0 (0.0%)	0 (0.0%)
Current smoker	35 (20.6%)*	63 (11.6%)*	19 (20.4%)*	76 (11.0%)*
Alcohol intake (g/day) median (IQR)			18.35 (28.74)	13.51 (26.98)
Calcium intake (mg/day) *M* (SD)			952.4 (377.1)	952.4 (377.1)
CCI				
0 (least comorbidity)	156 (91.8%)	471 (87.1%)	79 (84.9%)	508 (73.4%)
1	11 (6.5%)	48 (8.9%)	10 (10.8%)	100 (14.5%)
2	3 (1.8%)	21 (3.9%)	3 (3.2%)	59 (8.5%)
3	0 (0.0%)	1 (0.2%)	1 (1.1%)	17 (2.5%)
4	0 (0.0%)	0 (0.0%)	0 (0.0%)	7 (1.0%)
5	0 (0.0%)	0 (0.0%)	0 (0.0%)	0 (0.0%)
6	0 (0.0%)	0 (0.0%)	0 (0.0%)	1 (0.1%)
Medication use				
Adrenal steroid hormones	3 (1.8%)	7 (1.3%)	0 (0.0%)	10 (1.4%)
Gonadal hormones	23 (13.5%)	65 (12.0%)	1 (1.1%)	2 (0.3%)
Thyroid hormones or antithyroid agents	3 (1.8%)	13 (2.4%)	1 (1.1%)	4 (0.6%)
Agents affecting calcium or bone metabolism	1 (0.6%)	5 (0.9%)	0 (0.0%)	6 (0.9%)
Antidepressants	12 (7.1%)*	10 (1.8%)*	13 (14.0%)*	17 (2.5%)*
Lumbar spine BMD (g/cm^2^) *M* (SD)	1.226 (0.171)*	1.169 (0.190)*	1.235 (0.161)*	1.294 (0.198)*
Femoral neck BMD (g/cm^2^) *M* (SD)	0.975 (0.154)*	0.925 (0.155)*	1.001 (0.146)	0.998 (0.149)

*Note*: Data regarding medication use were based on self‐report. Medication classes were derived from the classification system in MIMS Online.^34^ Data regarding alcohol and calcium intake were not available for women at baseline, as the dietary questionnaire was not introduced until after the baseline assessment had been completed for this cohort. *p < 0.05.

Abbreviations: BMD, bone mineral density; CCI, Charlson Comorbidity Index; IQR, interquartile range; M, mean; SD, standard deviation.

^a^ Except where indicated.

### 3.2 Relationship between anxiety disorders and BMD

Table 2 shows the GEE regression models for the relationships between anxiety disorders and BMD at the lumbar spine and the femoral neck. For women, anxiety disorders were not significantly associated with BMD at either the lumbar spine (partial *η* ^2^ = 0.0003; *p* = 0.168) or the femoral neck (partial *η* ^2^ = 0.002; *p* = 0.720). For men, compared with the healthy group, the anxiety disorders group had lower BMD at both the lumbar spine (partial *η* ^2^ = 0.006; *p* = 0.018) and the femoral neck (partial *η* ^2^ = 0.006; *p* = 0.003). The interaction between group and weight (anxiety disorders group × weight) was significant for femoral neck BMD in men (partial *η* ^2^ = 0.004; *p* = 0.006). Otherwise, there were no significant interaction effects. In particular, the interaction between group and time (anxiety disorders group × time) was non‐significant for both sexes at both BMD sites, indicating that anxiety disorders were not associated with a significant difference in the rate of change in BMD over time.

**TABLE 2:** GEE regression models for BMD at the lumbar spine and femoral neck for women and men.

		Variables	Model‐adjusted regression coefficient	Partial *η* ^2^	95% Wald CI		Wald chi‐square (df)	*p* value
					Lower	Upper		
Women	Lumbar spine BMD	Anxiety disorders group	−0.013	0.0003	−0.031	0.005	1.901 (1)	0.168
		Time	−0.025	0.002	−0.033	−0.016	28.853 (1)	<0.001*
		Age at baseline (years)	−0.003	0.075	−0.004	−0.003	96.150 (1)	<0.001*
		Height (cm)	0.002	0.003	0.0004	0.004	5.501 (1)	0.019*
		Weight (kg)	0.003	0.075	0.002	0.003	52.340 (1)	<0.001*
		Socioeconomic status	0.006	0.002	0.001	0.012	4.647 (1)	0.031*
		Physical activity levels	−0.005	0.001	−0.013	0.004	1.181 (1)	0.277
		Current smoker	0.00009	0.0001	−0.018	0.018	0.000 (1)	0.992
		Alcohol intake (g/day)	0.0002	0.003	−0.0004	0.001	0.395 (1)	0.530
		Calcium intake (mg/day)	0.000006	0.001	−0.00002	0.00003	0.278 (1)	0.598
		CCI	0.002	0.001	−0.008	0.013	0.209 (1)	0.648
		Current use of adrenal steroid hormones	−0.028	0.0001	−0.075	0.018	1.420 (1)	0.233
		Current use of gonadal hormones	0.020	0.002	−0.007	0.047	2.029 (1)	0.154
		Current use of thyroid agents	0.029	0.004	−0.013	0.071	1.863 (1)	0.172
		Current use of calcium‐affecting agents	−0.011	0.012	−0.055	0.033	0.248 (1)	0.619
		Current use of antidepressants	0.002	0.00006	−0.019	0.022	0.025 (1)	0.875
	Femoral neck BMD	Anxiety disorders group	−0.003	0.002	−0.018	0.013	0.129 (1)	0.720
		Time	−0.042	0.014	−0.049	−0.035	147.575 (1)	<0.001*
		Age at baseline (years)	−0.005	0.224	−0.005	−0.004	334.744 (1)	<0.001*
		Height (cm)	0.001	0.00007	−0.0004	0.002	1.883 (1)	0.170
		Weight (kg)	0.003	0.186	0.003	0.004	168.297 (1)	<0.001*
		Socioeconomic status	0.004	0.004	−0.00006	0.009	3.734 (1)	0.053
		Physical activity levels	−0.011	0.014	−0.018	−0.004	9.701 (1)	0.002*
		Current smoker	0.006	0.000004	−0.012	0.025	0.436 (1)	0.509
		Alcohol intake (g/day)	−0.001	0.0002	−0.001	−0.0001	6.375 (1)	0.012*
		Calcium intake (mg/day)	0.00002	0.001	−0.000002	0.00003	3.1832404 (1)	0.074
		CCI	0.001	0.00004	−0.006	0.007	0.034 (1)	0.854
		Current use of adrenal steroid hormones	−0.010	0.00007	−0.043	0.022	0.378 (1)	0.539
		Current use of gonadal hormones	0.015	0.0002	−0.006	0.036	1.977 (1)	0.160
		Current use of thyroid agents	0.048	0.012	0.019	0.076	10.827 (1)	0.001*
		Current use of calcium‐affecting agents	−0.003	0.008	−0.035	0.030	0.026 (1)	0.871
		Current use of antidepressants	−0.001	0.0003	−0.016	0.014	0.008 (1)	0.929
Men	Lumbar spine BMD	Anxiety disorders group	−0.040	0.006	−0.074	−0.007	5.551 (1)	0.018*
		Time	0.053	0.016	0.041	0.066	70.569 (1)	<0.001*
		Age at baseline (years)	0.002	0.028	0.001	0.003	18.224 (1)	<0.001*
		Height (cm)	0.003	0.005	0.0002	0.005	4.377 (1)	0.036*
		Weight (kg)	0.002	0.025	0.0004	0.003	6.752 (1)	0.009*
		Socioeconomic status	−0.007	0.001	−0.014	−0.0003	4.132 (1)	0.042*
		Physical activity levels	0.001	0.001	−0.010	0.011	0.011 (1)	0.917
		Current smoker	−0.028	0.001	−0.059	0.003	3.055 (1)	0.080
		Alcohol intake (g/day)	0.0002	0.002	−0.0002	0.001	0.675 (1)	0.411
		Calcium intake (mg/day)	0.000004	0.001	−0.00002	0.00003	0.104 (1)	0.747
		CCI	0.008	0.001	−0.003	0.018	2.173 (1)	0.140
		Current use of adrenal steroid hormones	−0.064	0.004	−0.144	0.016	2.426 (1)	0.119
		Current use of gonadal hormones	−0.053	0.000004	−0.121	0.015	2.353 (1)	0.125
		Current use of thyroid agents	0.069	0.003	−0.050	0.188	1.278 (1)	0.258
		Current use of calcium‐affecting agents	−0.021	0.011	−0.082	0.040	0.461 (1)	0.497
		Current use of antidepressants	−0.018	0.002	−0.058	0.022	0.773 (1)	0.379
	Femoral neck BMD	Anxiety disorders group	−0.196	0.006	−0.325	−0.067	8.853 (1)	0.003*
		Time	−0.059	0.039	−0.068	−0.051	197.135 (1)	<0.001*
		Age at baseline (years)	−0.003	0.083	−0.004	−0.002	98.414 (1)	<0.001*
		Height (cm)	−0.0002	0.00003	−0.002	0.001	0.070 (1)	0.791
		Weight (kg)	0.003	0.079	0.003	0.004	105.045	<0.001*
		Socioeconomic status	−0.005	0.002	−0.010	−0.001	4.796 (1)	0.029*
		Physical activity levels	−0.014	0.006	−0.021	−0.006	13.650 (1)	<0.001*
		Current smoker	0.001	0.0001	−0.018	0.020	0.010 (1)	0.919
		Alcohol intake (g/day)	0.0001	0.001	−0.0002	0.0004	0.657 (1)	0.418
		Calcium intake (mg/day)	0.00001	0.003	−0.000003	0.00003	2.475 (1)	0.116
		CCI	0.003	0.00008	−0.003	0.010	0.997 (1)	0.318
		Current use of adrenal steroid hormones	−0.017	0.004	−0.056	0.021	0.758 (1)	0.384
		Current use of gonadal hormones	−0.044	0.0003	−0.091	0.004	3.262 (1)	0.071
		Current use of thyroid agents	0.027	0.0005	−0.042	0.096	0.604 (1)	0.437
		Current use of calcium‐affecting agents	−0.005	0.012	−0.041	0.031	0.064 (1)	0.800
		Current use of antidepressants	−0.006	0.001	−0.028	0.016	0.307 (1)	0.579
		Anxiety disorders group × Weight	0.002	0.004	0.001	0.004	7.418 (1)	0.006*

*Note*: Time was an ordinal variable in the analysis with the baseline time point as the reference class. Socioeconomic status, CCI and physical activity levels were treated as continuous variables. ‘Calcium‐affecting agents’ were agents affecting calcium or bone metabolism and ‘thyroid agents’ were thyroid hormones or antithyroid agents according to the MIMS Online classification system.^34^ *p < 0.05.

Abbreviations: BMD, bone mineral density; CCI, Charlson Comorbidity Index; CI, confidence interval.

### 3.3 Exploratory subgroup analysis

Given the findings that anxiety disorders were associated with reduced lumbar spine and femoral neck BMD in men, an exploratory subgroup analysis was performed for the male cohort. In this analysis, men with a lifetime history of a comorbid mood disorder were excluded from the analysis. There were 42 men with a history of an anxiety disorder who did not have a history of a comorbid mood disorder. Table 3 shows the GEE regression models for the relationships between anxiety disorders and lumbar spine and femoral neck BMD in the subgroup. Anxiety disorders were not significantly associated with BMD at either the lumbar spine (partial *η* ^2^ = 0.00005; *p* = 0.562) or the femoral neck (partial *η* ^2^ = 0.001; *p* = 0.663). There were no significant interaction effects.

**TABLE 3:** GEE regression models for BMD at the lumbar spine and the femoral neck for exploratory subgroup analysis.

Variables		Model‐adjusted regression coefficient	Partial *η* ^2^	95% Wald CI		Wald chi‐square (df)	*p* value
				Lower	Upper		
Lumbar spine BMD	Anxiety disorders group	−0.016	0.00005	−0.070	0.038	0.336 (1)	0.562
	Time	0.055	0.016	0.042	0.068	70.706 (1)	<0.001*
	Age at baseline (years)	0.002	0.027	0.001	0.003	14.718 (1)	<0.001*
	Height (cm)	0.002	0.005	−0.00008	0.005	3.603 (1)	0.058
	Weight (kg)	0.002	0.028	0.0003	0.003	6.082 (1)	0.014*
	Socioeconomic status	−0.008	0.001	−0.016	−0.001	5.041 (1)	0.025*
	Physical activity levels	0.00007	0.001	−0.011	0.011	0.0001 (1)	0.991
	Current smoker	−0.028	0.001	−0.063	0.006	2.618 (1)	0.106
	Alcohol intake (g/day)	0.0003	0.004	−0.0001	0.0008	2.220 (1)	0.136
	Calcium intake (mg/day)	0.000008	0.002	−0.00002	0.00004	0.405 (1)	0.525
	CCI	0.009	0.001	−0.002	0.019	2.510 (1)	0.113
	Current use of adrenal steroid hormones	−0.048	0.003	−0.130	0.034	1.337 (1)	0.248
	Current use of gonadal hormones	−0.054	0.00003	−0.122	0.013	2.467 (1)	0.116
	Current use of thyroid agents	0.071	0.004	−0.051	0.194	1.301 (1)	0.254
	Current use of calcium‐affecting agents	−0.025	0.009	−0.094	0.043	0.523 (1)	0.469
	Current use of antidepressants	−0.015	0.002	−0.073	0.043	0.260 (1)	0.610
Femoral neck BMD	Anxiety disorders group	−0.007	0.001	−0.039	0.025	0.189 (1)	0.663
	Time	−0.060	0.044	−0.069	−0.052	188.337 (1)	<0.001*
	Age at baseline (years)	−0.003	0.083	−0.004	−0.002	94.737 (1)	<0.001*
	Height (cm)	−0.0002	0.00003	−0.002	0.001	0.042 (1)	0.837
	Weight (kg)	0.003	0.104	0.003	0.004	110.043	<0.001*
	Socioeconomic status	−0.006	0.002	−0.011	−0.001	6.526 (1)	0.011*
	Physical activity levels	−0.015	0.010	−0.023	−0.007	14.777	<0.001*
	Current smoker	0.001	0.00005	−0.020	0.021	0.006 (1)	0.940
	Alcohol intake (g/day)	0.0002	0.001	−0.0001	0.0005	1.132 (1)	0.287
	Calcium intake (mg/day)	0.00001	0.003	−0.000003	0.00003	2.636 (1)	0.104
	CCI	0.004	0.0001	−0.003	0.010	1.109 (1)	0.292
	Current use of adrenal steroid hormones	−0.022	0.005	−0.063	0.019	1.083 (1)	0.298
	Current use of gonadal hormones	−0.040	0.0004	−0.082	0.002	3.403 (1)	0.065
	Current use of thyroid agents	0.032	0.002	−0.034	0.098	0.890 (1)	0.345
	Current use of calcium‐affecting agents	−0.009	0.010	−0.048	0.030	0.212 (1)	0.645
	Current use of antidepressants	−0.011	0.001	−0.039	0.017	0.638 (1)	0.424

*Note*: Time was an ordinal variable in the analysis with the baseline time point as the reference class. Socioeconomic status, CCI and physical activity levels were treated as continuous variables. ‘Calcium‐affecting agents’ were agents affecting calcium or bone metabolism and ‘thyroid agents’ were thyroid hormones or antithyroid agents according to the MIMS Online classification system.^34^ *p < 0.05.

Abbreviations: BMD, bone mineral density; CI, confidence interval; CCI, Charlson Comorbidity Index.

## 4 DISCUSSION

As far as we are aware, this is the first study to investigate the relationship between clinician‐diagnosed anxiety disorders and BMD. A lifetime history of a DSM‐IV‐TR anxiety disorder was associated with reduced BMD at both the lumbar spine and femoral neck in men. These associations were independent of differences in age, height, weight, socioeconomic status, physical activity levels, smoking, alcohol intake, calcium intake, medical history and use of medications known to affect bone health. The effect sizes were moderate, with a partial *η* ^2^ of 0.006 for both associations. However, when we performed an exploratory subgroup analysis that excluded participants with a history of a comorbid mood disorder, the associations became non‐significant. Anxiety disorders were not associated with significant differences in BMD in women. There was also no observed relationship between anxiety disorders and the rate of change in BMD in either sex. Therefore, when the effect of comorbid mood disorders was accounted for, anxiety disorders were not associated with either BMD or its rate of change in women or men.

The first question that arises regarding these findings concerns the factors responsible for the associations found between anxiety disorders and reduced BMD in men. Given the established relationship between depression and low BMD, it is possible that these associations were mediated by comorbid depressive disorders. Anxiety disorders are highly comorbid with depressive disorders. A 2011 Dutch study involving a large clinical sample found that, of persons with a current DSM‐IV‐TR anxiety disorder, 61% and 79% also met criteria for a current or lifetime history of major depressive disorder, respectively.^35^ The possibility of a mediating role for depression is supported by the lack of significant associations between anxiety disorders and BMD in the exploratory subgroup analysis. However, there were only 42 participants in the anxiety disorders group in this analysis and it is possible that the analysis was under‐powered. If the associations between anxiety disorders and BMD in men were mediated by comorbid depression, it is unclear why depression did not have a similar effect, and produce similar associations, in female participants.

An alternative possibility is that anxiety disorders impact adversely on BMD in men independently of the effects of comorbid depression. There are various mechanisms by which these disorders could potentially affect bone metabolism. These mechanisms include altered glucocorticoid secretion,^36^, ^37^ activation of immune and inflammatory pathways^38^, ^39^ and premature ageing.^40^ Converging evidence suggests that anxiety disorders may be associated with an acceleration of the ageing process. A recent study involving over 300,000 participants found differences between participants with anxiety disorders and healthy controls on a range of age‐related physiological measures, including blood pressure, heart rate, hand‐grip strength and body composition measures.^41^ Relevantly for our purposes, anxiety disorders were associated with lower estimated heel BMD in men (standardised mean difference (SMD) = −0.067, 95% CI −0.088 to −0.047) but not in women.

Anxiety disorders may also cause behavioural changes that impact on BMD. There is evidence that these disorders are associated with increased tobacco smoking and alcohol consumption, physical inactivity and poor dietary intake, which are all risk factors for low BMD.^42^, ^43^, ^44^, ^45^, ^46^, ^47^ In this analysis, the effects of smoking, alcohol intake, calcium intake and self‐reported physical activity levels were adjusted for. However, this adjustment may not have fully captured the effects of the harmful behaviours associated with anxiety disorders.

If anxiety disorders impact adversely on BMD in men via biological or behavioural mechanisms, it is important to consider why these impacts do not also occur in women. A priori, there is no reason why, for instance, altered glucocorticoid secretion should cause reduced BMD in men but not in women. Interestingly, our findings replicate the results of two previous large studies investigating the relationship between anxiety disorders (defined using self‐report measures) and BMD or estimated BMD.^24^, ^41^ Both studies found that anxiety disorders were associated with reduced BMD in men but not in women. These findings contrast with those of depression studies, which have found that depression is associated with reduced BMD in both sexes.^12^, ^48^ They raise the question of whether there are sex‐based differences in the effects of anxiety disorders on bone tissue. There are well‐established differences between the sexes in the epidemiology of anxiety disorders. Women are approximately twice as likely as men to suffer from these disorders and experience greater disability due to them.^49^ These epidemiological differences are believed to be partly due to differences between the sexes in the neural circuits underlying anxiety and fear responses.^50^ One physiological system that could potentially contribute to sex‐based differences in the effects of anxiety disorders on bone health is the hypothalamic–pituitary–gonadal (HPG) axis. Gonadal hormones play an important role in the regulation of bone tissue and are also involved in regulating anxiety and fear responses.^51^ We suggest that future studies of the relationship between anxiety disorders and BMD include measurement of HPG axis hormones so that their contribution to this relationship and any sex‐based differences in it can be explored.

This study had several important strengths. First, it had a prospective design and was able to investigate longitudinal relationships between anxiety disorders and BMD. Secondly, the study population was large and representative of the general population. Thirdly, the analysis adjusted for the effects of most established risk factors for osteoporosis and other possible confounders. Fourthly, BMD was measured using DXA at two clinically relevant sites, the lumbar spine and femoral neck. Fifthly, participants were assessed for a lifetime history of an anxiety disorder by a clinician using the SCID‐I/NP, the gold standard for psychiatric diagnosis. The study also had some limitations. First, inquiring about past psychiatric symptoms may be associated with a recall bias.^52^ Secondly, there was only a 5‐year gap between the time‐points at which the SCID‐I/NP was administered for women and a 10‐year gap for men. BMD decreases very slowly after the age of peak bone mass and these time periods may have been insufficient to detect significant differences in the rate of change in BMD over time. Thirdly, defining the healthy group as all GOS participants with no Axis I psychiatric disorders on the SCID‐I/NP may have inflated the differences between the anxiety disorders group and the healthy group, since the former group could include participants with non‐anxiety psychiatric comorbidities whereas the latter group could not. Fourthly, not all potential confounders were controlled for in the analysis. For instance, vitamin D levels were not adjusted for. Finally, the study utilised the diagnostic criteria in DSM‐IV‐TR, which has now been replaced by the fifth edition of the DSM (DSM‐5). In DSM‐5, the names and diagnostic criteria for the anxiety disorders have been updated and PTSD, acute stress disorder and OCD are no longer included in the anxiety disorders chapter.

In summary, this study found that men with a lifetime history of a DSM‐IV‐TR anxiety disorder had lower BMD in the lumbar spine and femoral neck compared with healthy controls. These associations may have been mediated by comorbid depressive disorders, as they became non‐significant when participants with a history of a mood disorder were excluded from the analysis. We hope that our findings will spur further research into the bone health of people suffering from anxiety disorders and other mental health conditions.

## FUNDING INFORMATION

Michael Berk is supported by a NHMRC Senior Principal Research Fellowship (1156072). Lana Williams is supported by a NHMRC Emerging Leadership Fellowship (1174060).

## CONFLICT OF INTEREST STATEMENT

The authors declare no conflict of interest.

### PEER REVIEW

The peer review history for this article is available at https://www.webofscience.com/api/gateway/wos/peer‐review/10.1111/acps.13563.

## Data Availability

The data that support the findings of this study are available on request from the corresponding author. The data are not publicly available due to privacy or ethical restrictions.
